# Microstructure and Intrinsic Strain of Nanocrystals in Ferroelectric (Na,K)NbO_3_ Nanofibers

**DOI:** 10.3390/nano12091541

**Published:** 2022-05-02

**Authors:** Alexander M. Grishin

**Affiliations:** 1 Division of Electronics and Embedded Systems, School of Electrical Engineering and Computer Science, KTH Royal Institute of Technology, SE-164 40 Stockholm, Sweden; grishin@kth.se; Tel.: +46-70-771-4176; 2 INMATECH Intelligent Materials Technology, SE-127 51 Skärholmen, Sweden

**Keywords:** nanofibers, lead-free, biocompatibility, ferroelectricity, crystal lattice parameters, fibers morphology, nanocrystals intrinsic strain

## Abstract

Densely woven highly crystallized biocompatible sodium–potassium niobate Na_0.35_K_0.65_NbO_3_ fibers with an average diameter of 100–200 nm and several hundreds of microns in length were sintered by the sol–gel calcination-assisted electrospinning technique. X-ray diffraction (XRD) and high-resolution transmission electron microscopy (TEM) confirmed preferential cube-on-cube [001] orientation of nanocrystals within the fiber’s body, separated by a low angle grain boundary. The Williamson–Hall method was employed to analyze the broadening of XRD reflections and to accurately determine the size and intrinsic strain of nanocrystal fiber aggregates. The main objective of this article is to test the potential capacity of direct XRD analysis to noninvasively control crystallite size and lattice distortion in core-shell coaxial nanofibers.

## 1. Introduction

The story of sodium–potassium niobate ceramics ((Na,K)NbO_3_, hereinafter NKN) was started in 1949 by Matthias, who, in Bell Labs, grew perovskite NKN single crystals, and evidenced their piezoelectricity, birefringence, the presence of dielectric hysteresis loop, and polymorphic phase transition below their Curie points [1]. Over the next five decades, work on NKN was mainly dedicated to the refinement of a phase diagram of continuous solid solution Na*_x_*K_1−*x*_NbO_3_, proof of ferroelectricity, and achievement of the maximum value of piezoelectric constant *d*_33_ = 160 pC/N [pm/V] occurred at the morphotropic phase boundary *x* = 0.5 [2,3,4,5]. The next important step in the exploration of NKN occurred in 1998. Thorough toxicology tests ascertained biocompatibility of NKN materials; no bacterial products (endotoxin) appear and the presence of NKN ceramics does not negatively affect the long-term viability of human monocytes. Ferroelectric NKN ceramics were FDA-approved (the U.S. Food and Drug Administration) and patented as a biocompatible material for implants [6].

The volatility of sodium and potassium has impeded the high yield fabrication of NKN films for a long time. Firstly, NKN films were cathode sputtered in 1988 [7]. Nevertheless, high-performance NKN films were grown by RF-magnetron sputtering [8,9,10] and the pulsed laser deposition (PLD) technique [11,12] only in the late 1990s and early 2000s. Since 2000, the number of papers, especially on Li and Ta substituted NKN, grows exponentially (e.g., [13,14]). In succeeding years, further attempts were made to demonstrate the applicability of thin NKN films for biocompatible radio-frequency readable (RFID) barcode and pacemaker pressure sensors, voltage tunable microwave varactors, acoustoelectric delay line, and waveguides for integrated optics [15,16,17] (see also the details and references in [18]). Nowadays, extensive research has been conducted to reveal the dopant–structure–functional property relationship in multi-element-doped NKN ceramics [19,20].

Recently, single-crystalline NKN nanofibers endow the portfolio of lead-free biocompatible strongly ferroelectric materials. Dense homogeneous NKN nanofiber fabric was sintered by a sol–gel calcination-assisted electrospinning technique [21,22]. Electrospinning is a simple and effective method for fabricating ultrathin threads. It was patented in 1934 [23]. Combined with a sol–gel calcination, this technique requires neither catalysts nor templates, yields highly crystalline nanofibers, and has attracted continuously growing interest since the mid-1990s [24]. Prepared NKN nanofibers can withstand without a breakdown of the electric field as high as 0.3 MV/cm, possess piezoelectric coefficient *d*_33_ = 75.8 pC/N, have reproducible bipolar resistive switching with the ON–OFF resistance ratio as high as 2 × 10^4^ [25], and have bright photoluminescence in Er-doped fibers [18]. The newest discovery of the magneto-electric effect in core-shell fibers of hexagonal ferrites and ferroelectric lead zirconate/barium titanates disclosed new functional properties and potential applicability of multiferroic nanofibers [26,27]. Ferromagnetic resonance (FMR) in coaxial Y_3_Fe_5_O_12_ core-Na_0.5_K_0.5_NbO_3_ shell nanofibers [28] and potential encapsulation of Gd_2_O_3_ threads [29] inside the NKN sheath promise new applications of biocompatible NKN-coated coaxial fibers to serve as potential agents for microwave magnetic hyperthermia, multifunctional 3D magnetic field/tensile stress sensors, energy harvesting nanogenerators, multimodal magnetic resonance imaging (MRI) and neutron capture therapy (see also [30,31,32,33]).

Although our first attempt to fabricate multiferroic coaxial Y_3_Fe_5_O_12_ core-Na_0.5_K_0.5_NbO_3_ shell nanofibers was attended with success, it also revealed a serious problem. To guarantee a single-phase content of two chemically and structurally dissimilar substances, it demands different calcination temperatures: 1100 °C for Y_3_Fe_5_O_12_ [34] and 800 °C for (Na,K)NbO_3_ [21]. During calcination, a polymer binder vaporizes and two adverse materials in coaxial threads, tightly adherent to each other, experience very strong shrinkage. The resulting elastic stress gives rise to a twice stronger deformation of NKN crystalline lattice due to a big difference in Young’s modulus, E* = 100 GPa in (Na,K)NbO_3_ [35] and 206 GPa in Y_3_Fe_5_O_12_ ferrite [36]. This circumstance brings specific peculiarity to the phenomenon of crystal size confinement for coaxial nanofibers with a high surface-area-to-volume ratio.

Static magnetic and ferromagnetic resonance (FMR) properties in ferrite fibers are governed by their shape and built-in magnetocrystalline anisotropy field (planar or uniaxial) [26,27,28,34]. In ferroelectric fibers, the piezoelectric coefficient *d*_33_ is strongly anisotropic varying from 75.8 in the out-of-fiber axis to 18.3 pC/N in the on-axis oriented ferroelectric domains [22]. All the above-mentioned factors, to a relatively high degree, depend upon the intrinsic strain of the correspondent material. Therefore, to reach the ultimate single-crystalline ferromagnetic and ferroelectric properties, one should minimize the microstrain appearing in multiferroic fibers.

The accurate control of desirable fibers’ crystallinity is an important precondition for the electrospinning process of coaxial nanofibers. Besides a standard examination of a phase content and predominant crystals growth, a long-established direct XRD method of calculating the integral breadths of Bragg reflections with a certain accuracy can answer most of the questions regarding the crystal size confinement. This paper constitutes the first report on the employment of this approach to characterize single-crystalline ferroelectric nanofibers. Herein, we compare XRD and electron microscopy data on the size, orientation and strain of nanocrystals in electrospun (Na,K)NbO_3_ fibers.

## 2. Experimental Section

Synthesis of highly crystalline (Na,K)NbO_3_ nanofibers by a sol–gel calcination-assisted electrospinning technique was described earlier [21]. In brief, NKN precursor solution contained a mixer of sodium NaO_2_C_2_H_3_×H_2_O (Thermo Scientific^TM^, 99.9%, Waltham, MA, USA) and potassium KO_2_C_2_H_3_×H_2_O (Thermo Scientific^TM^, 99%) acetates and 2-methoxyethanol C_3_H_8_O_2_ (Sigma-Aldrich^®^, 99.8%, St. Louis, MO, USA). To prepare the solution for electrospinning, niobium ethoxide C_10_H_25_NbO_5_ (Thermo Scientific^TM^, 99.9%) was dissolved in acetyl–acetone C_5_H_8_O_2_ (Thermo Scientific^TM^, 99%, as a chelating agent), mixed with polyvinylpyrrolidone (PVP, Alfa Aesar, Ward Hill, MA, USA; 0.035 g/mL, as a binder), and added to the NKN sol. Viscous polymer jet was ejected from a syringe pump that feeds PVP/NKN solution at a constant rate of 0.5 mL/h in electric field 1.8 kV/cm between metallic needle and aluminum foil collector. Bead-free nanofibers were dried at 100 °C in nitrogen atmosphere for 12 h and annealed at 800 °C for 1 h in air.

Crystallized fibers became very fragile thus to prepare samples for optical, electron microscopy and XRD examinations, a porous 50 μm thick layer of as-spun randomly woven fibrous material was folded several times to make a flake. Such 4–8 multilayered flakes shrink at calcinations to the lateral size of 2 × 2 mm^2^. A three-dimensional laser scanning microscope *Keyence* VK-9710 was used to visualize surface morphology of fibers’ fabric. Scanning electron microscopy (SEM) images were collected with a field emission microscope *JEOL JSM*-7500FA while the high-resolution transmission electron microscopy scans were performed with *JEOL JEM* 2011 (TEM, 200 keV). *Siemens* D-5000 powder X-ray diffractometer was used to display phase content and crystalline structure of nanofibers. The full width at half maximum (FWHM, as narrow as 0.02°) of the rocking curve for a standard Ca,Mg,Zr:Gd_3_Ga_5_O_12_ (111) single crystal substrate is considered as a measure of instrumental broadening. Piezoresponse recorded by *Asylum Research* MFP-3D atomic force microscope (AFM) with a PtIr-coated tip of Si cantilever confirmed ferroelectricity in individual NKN fibers clamped onto iridium coated Si wafer. Memristor-type resistance switching in Au/NKN/Pt diode cell was uncovered from current–voltage *I–V* characteristics traced with a *Keithley* 2410 SourceMeter (Solon, OH, USA).

## 3. Results and Discussion

### 3.1. Crystallized Fibers’ Morphology

**Figure 1:**
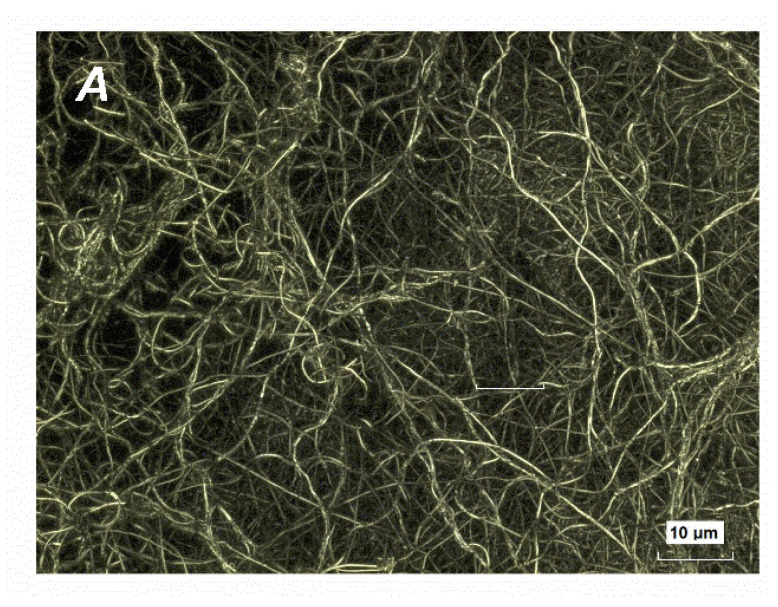

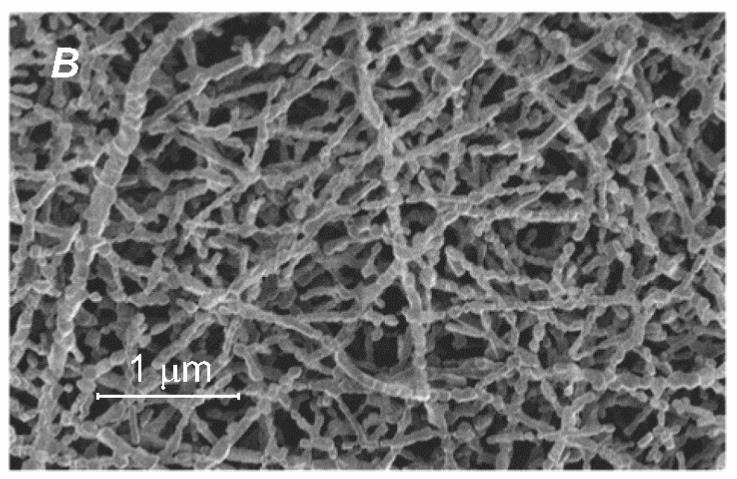
(Na,K)NbO_3_ nanofibers calcined at 800 °C in air. (**A**) Large depth-of-field optical image captured by 3D laser scanning microscope *Keyence* VK-9710; (**B**) SEM image copied by means of *JEOL JSM*-7500FA field emission scanning electron microscope.

During annealing at 800 °C in air, due to the vaporization of the PVP binder, ejected viscous threads experience strong shrinkage and transform into densely woven crystallized fibers with an average diameter of 100–200 nm and several hundreds of microns in length. On the outer surface of a packed flake specimen, they are randomly in-plane oriented, see Figure 1.

**Figure 2:**
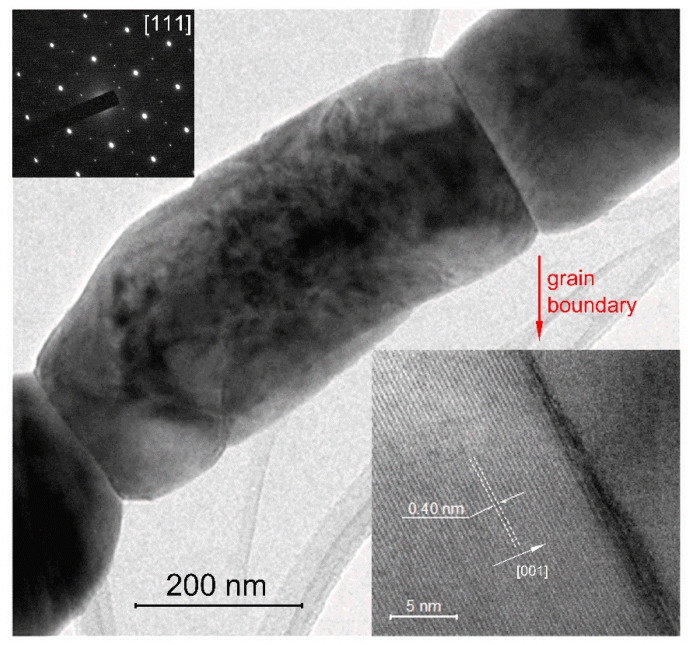
TEM images demonstrate two neck-separated single NKN nanocrystals. Upper inset presents selected area electron diffraction (SAED) pattern along [111] zone axes. High-resolution TEM image in lower inset shows the grain boundary between two adjacent nanocrystals.

TEM scans enable atomic resolution of NKN fibers crystallinity. As an example, Figure 2 exhibits a joint structure of two adjacent nanocrystals. The selected area electron diffraction (SAED) pattern testifies a monocrystalline fibers degree. The neck separates two neighboring nanocrystals. The TEM image in the lower inset in Figure 2 shows that the interior of the elongated nanocrystal is built up of perfectly ordered atomic (001) planes of 0.40 nm apart. They are oriented parallel to the grain boundary. Unfortunately, it was not possible to catch both single nanocrystals in focus to resolve a grain boundary structure. However, the XRD study that follows afterward, testifies a global predominant [001] crystal orientation in the NKN fabric composed of arbitrarily oriented fibers. It means that two neighboring grains most likely consist of [001] oriented crystals and are separated with a low-angle grain boundary.

### 3.2. Lattice Parameter

**Figure 3:**
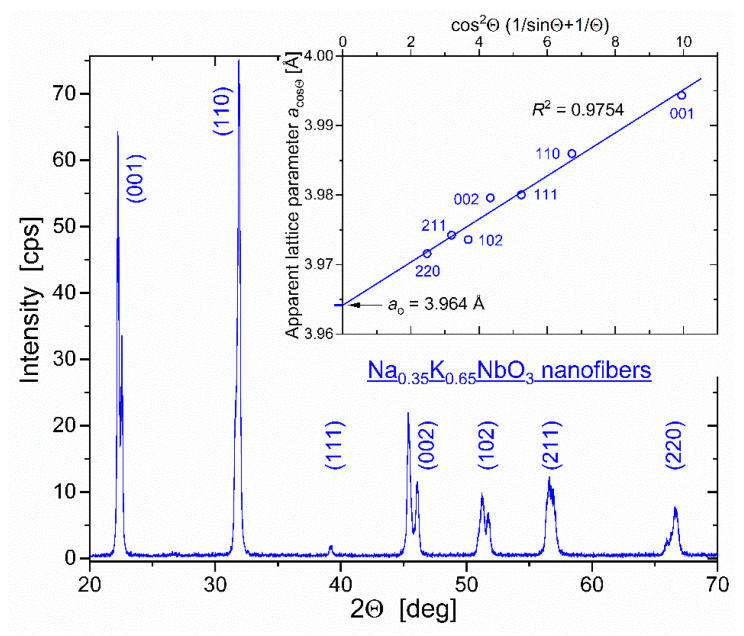
XRD patterns of NKN nanofibers recorded using a *Siemens* D-5000 powder diffractometer in Cu*K*_α__1_ radiation (λ = 1.5406 Å). Bragg reflections are notified by Miller indices for pseudo-cubic crystal unit cell Na_0.35_K_0.65_NbO_3_. Inset shows extrapolation of all the “apparent” parameters *a*_cosΘ_ to the “true” lattice parameter *a*_o_ = 3.964 Å using the Nelson–Riley Equation (1).

In Figure 3, the Θ–2Θ XRD scan confirms a single perovskite phase of NKN fibers calcined at 800 °C. The relative intensity ratios of (*hkl*) reflections indicate noticeable preferential NKN(001) orientation: *I*_001_/*I*_110_ = 0.86 in NKN fibers compared to 0.58 in an “ideal” Na_0.35_K_0.65_NbO_3_ (ICSD-38004) powder [37]. This observation conforms to [001]-directional cube-on-cube growth of nanocrystals visualized by TEM in Figure 2 and evidences low-angle-type grain boundaries between the two NKN neighbors.

**Figure 4:**
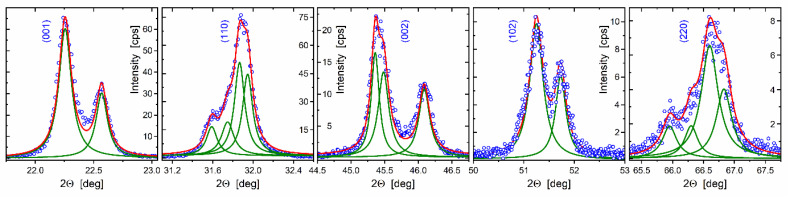
Circular symbols display enlarged Na_0.35_K_0.65_NbO_3_ Bragg reflections from the experimental XRD scan in Figure 3. Green color solid lines depict fitting Lorentzian components whereas summarizing curves are shown with a red color.

Five main XRD peaks were enlarged and deconvoluted by Lorentzian lines in Figure 4. They accord to the positions and the total number of all the Bragg manifolds characteristic for the monoclinic crystal system in Na_0.35_K_0.65_NbO_3_ (ICSD-38004) [37].

The “true” lattice parameter *a*_o_ was found by plotting in the inset of Figure 3 the “apparent” parameters *a*_cosΘ_ vs. diffraction angle 2Θ using the Nelson–Riley function [38]:

(1) $$\frac{a_{\cos\Theta }-a_{o}}{a_{o}}=A\;\cos^{2}\Theta \;\left(\frac{1}{\sin\Theta }+\frac{1}{\Theta }\right)$$

here, *a*_cosΘ_ is the interplane distance calculated from the apparent Bragg peak position at 2Θ. *A* is a fitting coefficient, the angle Θ in the second term 1/Θ in brackets is measured in radians. Circular symbols in the inset to Figure 3 display the “apparent” lattice parameters *a*_cosΘ_ obtained for the correspondent NKN (*hkl*) reflections. The highest left peak at 2Θ = 22.24 deg from the doubled (001) reflection gives *a* = 3.994 Å as (001) interplane distance. The true lattice parameter *a*_o_ = 3.964 Å we determine as an extrapolation of *a*_cosΘ_ to cosΘ → 0. Within the 0.04% accuracy, the ascertained value *a*_o_ coincides with the twice downsized $\left(a+b+c\right)/3$ averaged lattice parameter of monoclinic Na_0.35_K_0.65_NbO_3_ Bravais lattice (ICSD-38004) [37].

### 3.3. Ferroelectricity

**Figure 5:**
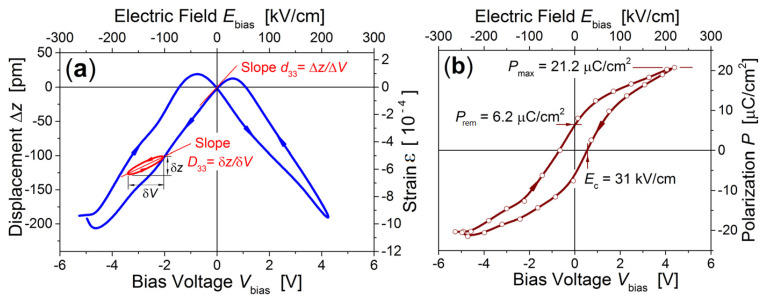
Electrostriction effect recorded with the *Asylum Research* MFP-3D AFM in individual NKN nanofiber. (**a**) Strain ε and displacement Δ*z* vs. bias voltage curve. Piezoelectric coefficient *d*_33_ is obtained as a slope of the butterfly loop at zero bias. Minor loop schematically shows how the “incremental” piezoelectric coefficient *D*_33_ can be experimentally revealed. (**b**) Hysteresis polarization *P–E* loop reconstructed from the butterfly displacement–voltage Δ*z*–*V*_bias_ curve. Reproduced from [22] with the permission of AIP Publishing.

Using piezoelectric force microscopy (PFM), we revealed the electrostriction effect in the NKN fibers. It displays itself as a nonlinear contraction of the transversal size of individual fiber under a high applied voltage. The butterfly-shaped loop in Figure 5a shows the displacement Δ*z* of AFM probe cantilever vs. triangular waveform bias voltage *V*_bias_ swept from − 5V to + 5V with the rate of 0.2 V/s. The longitudinal piezoelectric coefficient *d*_33_ = 56 pC/N is defined as a slope of Δ*z* vs. *V*_bias_ curve at zero bias. Compared to 160 pC/N in bulk NKN single crystals [1], the observed reduced values of piezoresponse in our fibers and *d*_33_ = 40 pC/N in 250 nm thick Na_0.5_K_0.5_NbO_3_ films [39], we rely upon the adherent clamping of ferroelectric materials onto the conducting substrate.

The ferroelectric hysteretic *P–E* loop we plotted in Figure 5b assumes quadratic dependence of electrostrictive strain upon the polarization: ε = *Q* × *P*^2^. To quantify *P* in [µC/cm^2^] units we chose the value of longitudinal electrostriction coefficient *Q* = 2.6 × 10^−2^ m^4^/C^2^ obtained for bulk Na_0.50_K_0.5_NbO_3_ ceramics [40]. Surprisingly, the main *P–E* loop characteristics: shape, coercive field *E*_c_ = 31 kV/cm, and the maximum polarization *P*_max_ = 21.2 µC/cm^2^ achieved in the field *E =* 220 kV/cm appeared to be very close to those we measured in pulsed laser Na_0.50_K_0.5_NbO_3_ films deposited onto the bulk Pt_80_Ir_20_ substrate [10].

### 3.4. Electrical Switching

**Figure 6:**
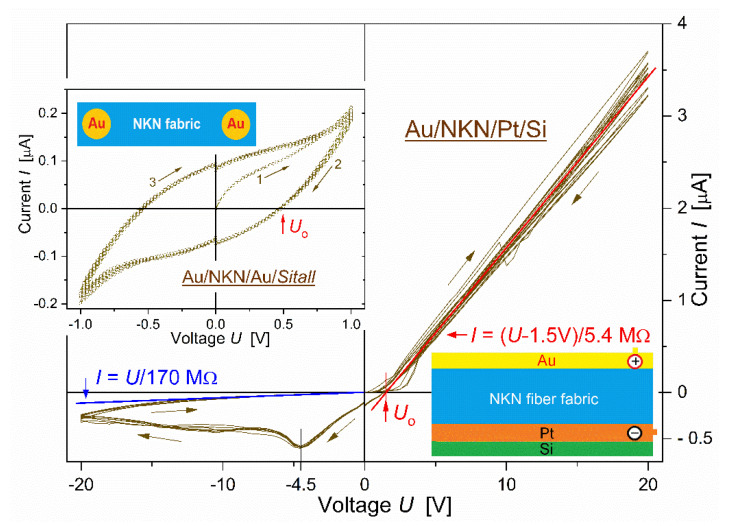
Current–voltage *I–V* characteristics traced with *Keithley* 2410 SourceMeter. Main frame–in the vertical Au/NKN(260 μm)/Pt/Si diode cell. Red straight line *I =* (*U* − 1.5 V)/5.4 MΩ is the average of multiple cycle *I–V* traces in the forward direction. Blue straight line *I =* *U*/170 MΩ fits the upward reversal *I–V* branch in the log–log scale (not shown). Left upper inset–enlarged part at low voltages in the planar Au/NKN(350 μm)/Au/*Sitall* cell onto dielectric substrate. In planar and vertical cells, remnant ferroelectric polarization nullifies the current in downward *I–V* branches at positive voltage *U*_o_ = 0.5 and 1.5 V, correspondingly.

The electrical properties of the tightly pressed NKN nanofiber fabric are described in Figure 6. The upper left inset presents the current–voltage *I–V* characteristic of a planar Au/NKN/Au cell. Two circular 0.95 mm diameter Ohmic Au contacts were thermally evaporated onto a 350 μm thick NKN filament specimen placed on the crystalline glass-ceramic *Sitall* substrate. Oppositely to the linear *I–V* curve at high voltages (not shown), at low applied voltages it has a hysteretic *clockwise*-directed character, highly reproducible within the ±1 V range. The initial upward branch **1** of the *I–V* curve starts at the origin. The downward curve **2** appears beneath the initial one due to the reduction of the effective electric field inside the ferroelectric material ***E***^(i)^ = ***E***^(e)^ − 4π$\hat{N}$***P***(***E***^(i)^), $\hat{N}$ is the tensor of depolarizing coefficients. At this descending branch of the *I–V* curve, the current goes to zero at positive voltage *U*_o_ = 0.5 V when the remnant polarization of the ferroelectric compensates for the applied external electric field: 4π$\hat{N}$***P***_rem_ = ***E***^(e)^.

The main frame of Figure 6 shows the *I–V* characteristic of the vertical Au/NKN/Pt cell onto the Si wafer. It consists of a top Au contact and a 260 μm thick NKN filament textile onto the Pt-coated Si as a bottom electrode. Such a cell acquires a strong rectification property. In the forward direction, the slope of the linear *I–V* characteristic yields a moderately *low resistance* of the NKN fabric of about 5.4 MΩ (resistivity 1.5 × 10^6^ Ω·cm). Applied −20 V reverse bias converts the Au/NKN/Pt cell from the low-to-high resistance state. To calculate resistance in the *high resistance* state, we fitted the upward reversal branch of the *I–V* curve in the log–log scale (not shown). The result is shown with a blue straight line *I* = *U*/170 MΩ in Figure 6. Reproducible bipolar resistive switching starts with a threshold voltage of −4.5 V, does not require an electroforming process and has a non-volatile character.

Vertical Au/NKN/Pt memristor exhibits ferroelectricity at low voltages. Similar to a planar Au/NKN/Au cell, the current in the downward *I–V* branch nullifies at the positive voltage *U*_o_ = 1.5 V. High porosity of the NKN nanofiber fabric leads to a greatly reduced value of induced ferroelectric polarization 4π$\hat{N}$***P***_rem_. This explains the narrow range of *U*_o_ voltages where the memory effect occurs. Resistive switching and a diode rectification property in the vertical Au/NKN/Pt cell are governed, correspondingly, by redox processes and the difference between the electrodes’ work functions (5.1 eV of Au and 5.65 eV of Pt). There is no resistive switching in the planar Au/NKN/Au cell. There, the hysteretic, unusually *clockwise*-directed, though the symmetrical *I–V* characteristic, displays the evidence of ferroelectricity.

### 3.5. Size and Intrinsic Strain of Nanocrystals

The presence of multiple XRD peaks of crystallized NKN fibers enables an accurate determination of both the size and intrinsic strain in nanocrystals that compose the nanofibers. A.R. Stokes and A.J.C. Wilson, perhaps, were among the first who suggested that the broadening of XRD reflections is produced by lattice strains and small particle sizes simultaneously [41,42]. G.K. Williamson and W.H. Hall put this proposition into practice, developing a new method to quantitatively ascertain the size and distortion of crystal aggregates [43]. Accordingly, both the crystalline size *D* and intrinsic strain *ε* govern the breadth of the X-ray diffraction peak. Therefore, the full width *B* at a half maximum (FWHM, measured in radians) of each peak that occurs at diffraction angle 2Θ includes two terms:

(2) $$B=\frac{0.9\;\lambda }{D\;\cos\Theta }+4\;\varepsilon \;\tan\Theta$$

The first one comes from a commonly used Scherrer formula containing a crystalline size *D*. The second term assumes that nanocrystals experience isotropic microstrain *ε*. Both these requisite parameters can easily be found by plotting data of *B*∙cosΘ vs. 4∙sinΘ for each diffraction peak as follows:

(3) $$B\;\cos\Theta =\frac{0.9\;\lambda }{D\;}+4\;\varepsilon \;\sin\Theta$$

Strictly speaking, the above presented so-called Williamson–Hall “uniform deformation model (UDM)” has no general character and cannot be applied to nanoparticles with an arbitrary crystalline structure. Nonetheless, we consider it applicable for sodium–potassium niobate nanofibers. Within the accuracy of employed XRD measurements, we cannot testify to the monoclinic crystal system of our NKN fibers. Everywhere through the manuscript, we describe (Na,K)NbO_3_ XRD spectra in pseudo-cubic representation. In reality, the difference of *a*, *b* and *c* parameters in monoclinic the Na_0.35_K_0.65_NbO_3_ (ICSD-38004) lattice is between 0.2% and 1.4% [37]. Therefore, with this precision, we treat the NKN crystal as a cubic one and employed the UDM model, which considers strain to be isotropic in nature.

**Figure 7:**
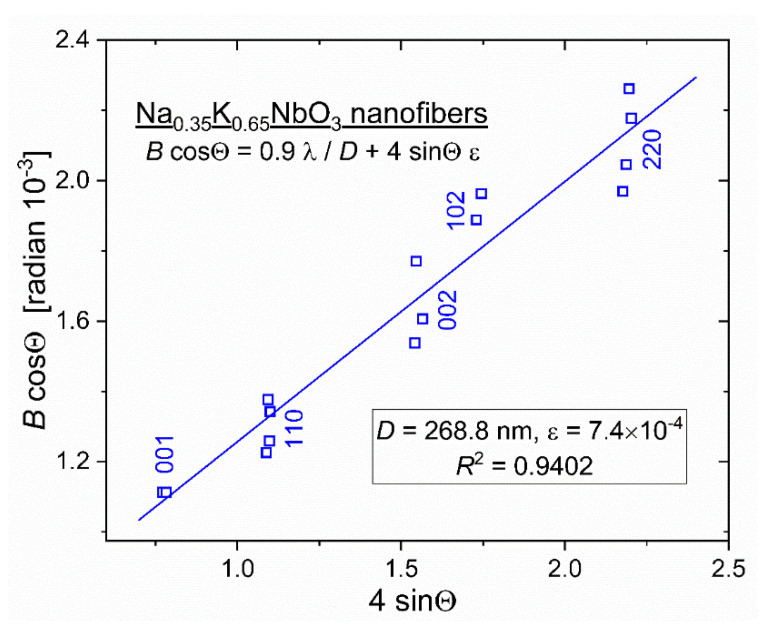
Williamson–Hall *B*∙cosΘ vs. 4∙sinΘ plot for five XRD reflections. The straight line with the correlation coefficient *R*^2^ = 0.9402 fits all the Bragg manifolds displayed in Figure 4.

Figure 7 shows the Williamson–Hall plot for the crystallized NKN fiber specimen. Square symbols mark positions 2Θ and FWHM values *B* for all the Lorentzian-deconvoluted manifolds, shown in Figure 4. Correction for instrumental breads, although negligibly less than *B*, was performed for every five Bragg reflections used for analysis. *B*∙cosΘ data were linearly fitted so that the slope of the fitting line yields the microstrain ε = 7.4 × 10^−4^ while the averaged crystal size *D* ≈ 270 nm is determined from its intersection with the ordinate axis at sinΘ → 0.

XRD-obtained values of the averaged crystal size *D* and the microstrain *ε* conform, respectively, to the high-resolution TEM image in Figure 2 and to as small as 0.04% difference between the determined in Figure 3 “true” lattice parameter *a*_o_ = 3.964 Å and the size of the Na_0.35_K_0.65_NbO_3_ Bravais lattice (ICSD-38004) [37].

We do believe that analysis of a broadening of multiple XRD reflections has the potential to reveal intrinsic strain and size of nanocrystals in on-fiber axis and out-off-fiber axis directions. For this purpose, fibers should be aligned with high precision. Then, a complete set of XRD scans (coupled θ–2θ, uncoupled θ+δθ–2θ, rocking curves and φ-scans) enable the comprehensive characterization of a fibers’ nanocrystal morphology and crystalline structure.

## 4. Conclusions

Comparison of crystalline characteristics of NKN filament fabric obtained by XRD and high-resolution TEM displays the potential capacity of direct XRD analysis to noninvasively control the size and intrinsic strain of nanocrystals in core-shell coaxial nanofibers. Further research is underway to employ the suggested method to early fabricated multiferroic coaxial Y_3_Fe_5_O_12_ core-(Na,K)NbO_3_ shell nanofibers [28]. We aim to determine the character (compressive or tensile) of intrinsic strain in chemically and structurally dissimilar, tightly adherent to each other, magnetically and electrically spontaneously ordered substances. Additionally, we compare the size of core ferrite Y_3_Fe_5_O_12_ nanocrystals determined from XRD and magnetic force microscopy (MFM) scans.

## Data Availability

Data are contained within the article.
